# Non-intrusive high throughput automated data collection from the home cage

**DOI:** 10.1016/j.heliyon.2019.e01454

**Published:** 2019-04-04

**Authors:** Fabio Iannello

**Affiliations:** Tecniplast SpA, Via I Maggio, 6, 21020 Buguggiate (VA), Italy

**Keywords:** Bioengineering, Bioinformatics, Cancer research, Genetics, Neuroscience, Physiology, Toxicology

## Abstract

Automated home cage monitoring represents a key technology to collect animal activity information directly from the home cage. The availability of 24/7 cage data enables extensive and quantitative assessment of mouse behavior and activity over long periods of time than possible otherwise. When home cage monitoring is performed directly at the home cage rack, it is possible to leverage additional advantages, including, e.g., partial (or total) reduction of animal handling, no need for setting up external data collection system as well as not requiring dedicated labs and personnel to perform tests. In this work we introduce a home cage-home rack monitoring system that is capable of continuously detecting spontaneous animal activity occurring in the home cage directly from the home cage rack. The proposed system is based on an electrical capacitance sensing technology that enables non-intrusive and continuous home cage monitoring. We then present a few animal activity metrics that are validated via comparison against a video camera-based tracking system. The results show that the proposed home-cage monitoring system can provide animal activity metrics that are comparable to the ones derived via a conventional video tracking system, with the advantage of system scalability, limited amount of both data generated and computational capabilities required to derive metrics.

## 1 Introduction

Automated home cage monitoring represents a key technology to measure spontaneous animal activity in rodents as it enables researchers to monitor animals over long periods of time without human intervention. Home cage monitoring systems have potential impact not only in extracting relevant information from scientific perspective, but they potentially provide support for improving animal welfare [1], [2], [3]. Monitoring home cages 24/7 enables the collection of data related to animal activity and behaviors, potentially spanning across several weeks, months or the entire animal life, which would otherwise be lost as simply not recorded. This provides a completely new set of information available to scientists (see e.g., discussion in [4], [5]). There exist several systems and technologies available for home cage monitoring [3], ranging from video cameras [6], [7], beam breaking systems [8] and force transducers [9] to other techniques based on passive infrared [10], piezoelectric [11] and microwaves [12]. Each technology has its own trade-offs, for example, camera-based systems potentially have the advantage of providing detailed images and the capability to observe animal locomotion and behaviors [6], [13], with main limitations being system scalability when the number of cages to observe becomes large (e.g., in terms of generated data), computational power and mechanical set up. Also other systems require ad-hoc mechanical set up, which could limit scalability and potentially require dedicated personnel for starting up the system [14].

Additional advantages of home cage monitoring systems arise when animal observation takes place directly at the *home cage rack*, including: enabling inherent high throughout data collection, as multiple cages can be monitored simultaneously; partially (or totally) reducing the need for both animal handling and using external testing devices with their dedicated labs (thus potentially reducing stress [15], [16], [17]) and long term 24/7 data collection.

In this paper, we analyze a non-intrusive *home cage-home rack* monitoring system, which is based on an electrical capacitance sensing technology [18]. The proposed home cage monitoring system is designed to gather 24/7 animal activity data directly from the home cage while keeping cages into conventional IVC racks that are compatible with the system (see Section 2.1). The goal of this paper is to introduce the reader to the proposed technology and describe relevant animal activity metrics as well as approaches used for their validation. Since this is the first paper describing the proposed home cage monitoring system in detail, we focus on individually-housed mice and consider the following animal activity metrics: distance walked, average speed, activation density and occupancy (see Sec. 2.4 for details). We propose a validation approach based on the comparison between the proposed system and video technology so that the same animal subjects are observed simultaneously with both systems (similarly to approaches proposed in, e.g., [7], [10], [19]). To compare the metrics derived with the two systems we resort to the Pearson correlation coefficient (see, e.g., [10], [14]).

## 2 Material & methods

### 2.1 Digital ventilated cage (DVC^®^) system

The home cage monitoring device presented in this paper is a commercial system known with the name of Digital Ventilated Cage (DVC^®^) manufactured by Tecniplast SpA (Buguggiate, Italy). This system is designed to collect information from individual ventilated cages (IVC) directly from the home cage rack. The system builds up on the top of a standard IVC rack by installing an electronic *sensing board* underneath each cage position. At the time of writing, the DVC^®^ system is either sold together with a fully equipped Tecniplast IVC rack, or alternatively can be installed (via a *retrofitting* procedure) on Tecniplast IVC racks of the series DGM for mice (green line) [18]. The latter is the only IVC rack backward compatible with the installation of the DVC^®^ system, no other IVC racks (either Tecniplast or from other vendors) are currently supported.

**Figure 1:**
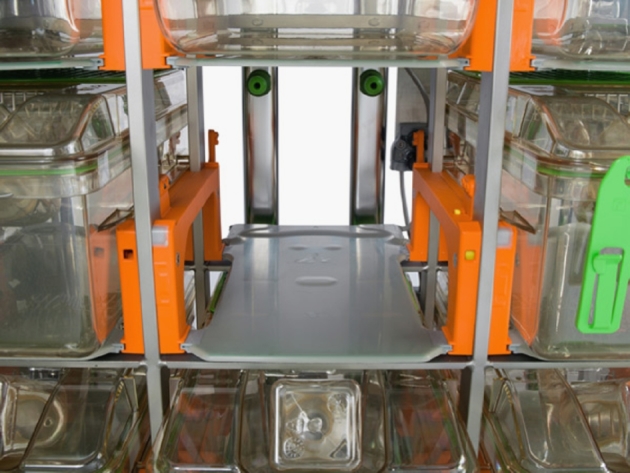
Capacitance sensing board installed at each cage position.

**Figure 2:**
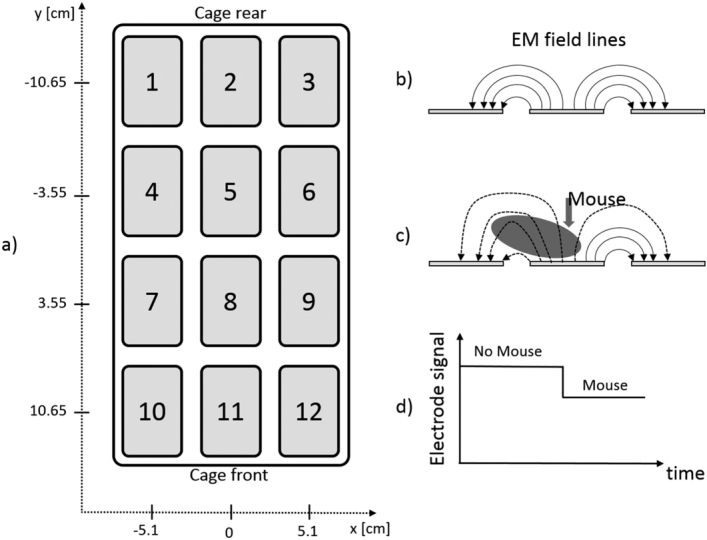
Panel a) shows the CST board with electrode numbering and the coordinates (x, y) of each electrode. Panel b) shows a side view of three electrodes together with a pictorial representation of the electromagnetic (EM) field lines (representing actual EM field lines is out of scope for this paper). Panel c) shows the effect of the presence of a mouse over an electrode that modifies the EM field lines distribution, thus causing a drop of the electrode signal (related to a change in electrical capacitance) as shown in panel d).

The proposed system is based on a sensing board that is mechanically connected to the rack without influencing conventional IVC cage operations (see Figure 1). The sensing board is composed of 12 electrodes connected to an integrated circuit that continuously measures their *electrical capacitance*. We refer to this sensing mechanism as capacitance sensing technology (CST). Since capacitance is influenced by the matter present in each electrode’s surrounding, its measurements are affected by the presence of, e.g., water and animals (see Figure 2). Note that, materials with high water content are characterized by large values of relative permittivity [20] (with respect to air), which in turns has a direct effect on capacitance (high relative permittivity means higher capacitance). Since mice are characterized by high water content, their movements performed while close to an electrode induce significant capacitance changes, and thus, by properly tracking these changes over time it is possible to monitor animal activity. Note that, capacitance remains substantially unchanged when material compositions around an electrode is unvaried. Additionally, the capacitance readings are affected by the presence of water (due to e.g., bottle leakage) or urine. However, animal activity occurs on a time scale substantially different than that of water leakage or urine and thus the two effects are easily separable. Furthermore, even when water/urine are present in an electrode surrounding (clearly not a flooded cage, but common amount of water/urine in a dirty cage) the capability of the system to discern animal movements is substantially unchanged. In fact, the presence of water/urine can change absolute capacitance readings, but not capacitance variations due to animal movements.

The CST board is installed at each cage position of the IVC rack, and is connected (via wire) to a power and data connection backbone infrastructure installed on the rack. Each rack, is then connected with a single cable to a dedicated computer, referred to as *master*, which provides both power and data connection to all electronic boards in the rack. Note that, no cables and sensors are connected directly to the cage, which is untouched by the *digitalization* of the rack (all sensing occurs externally to the cage and non-intrusively). The master collects raw data coming from all cage positions, with the option of both transferring them directly to a web-based software application or to a dedicated storage device for later data processing purposes. For this study, each electronic board is set up to collect capacitance measurements 4 times per second (i.e., 4 Hz) from each electrode of each cage, thus generating a set of 48 capacitance measurements per second (i.e., 12 electrodes sampled at 4 Hz). The amount of data generated by each board is approximately 2.5 MBytes per day per cage, which thus requires limited storage and computational capabilities. Note that, once the system is installed and configured, there is no need for human intervention to set up data collection as it continuously monitors and stores data from each cage position 24/7 automatically. Also power and data infrastructure are always available without requiring any manual set up.

### 2.2 Home cage video recording system

**Figure 3:**
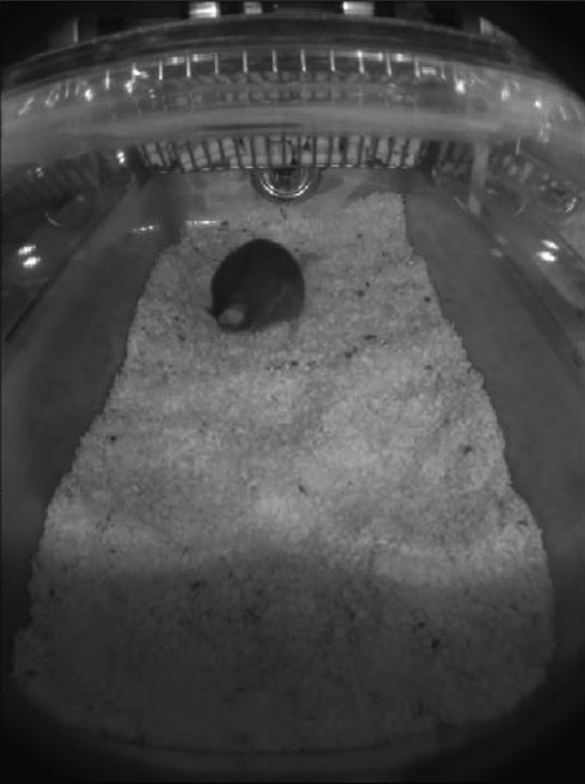
Screenshot of a frame captured by one of the video camera used in the test.

To validate the capacitance sensing technology proposed in this paper, we developed a home-cage camera-based monitoring system composed of a small portable computer connected to a video camera module and to the CST sensing board (placed underneath each cage under test). This set up enables synchronized start and stop acquisition between the camera and the CST board. The camera acquires videos at 10 frames/second with a resolution of 800x600 and it is also equipped with 4 infrared (IR) LED illuminators to enable visibility in low light and dark conditions. The camera module is designed so that non-intrusive continuous monitoring of home cage can be performed without impacting the conventional home cage setting (cage lid, water and food) while keeping the cage in the IVC rack and leaving animals undisturbed. This is obtained by placing the camera in the front part of the cage lid leading to a view that allows the monitoring of the whole cage floor as shown in Figure 3.

#### 2.2.1 Video processing software

We developed a dedicated video processing software able to identify the mouse position at each frame. Mouse movements are estimated from video frames based on a “blob” technique [21], where we exploited the fact that the background color (bedding and cage walls) was clearer than the mouse (C57BL/6J). Video frames were transformed into gray scale, and then converted into black and white based on a conveniently defined threshold. The result of the thresholding is to leave a black “blob” corresponding to the mouse body over a white background. The centroid of the black blob is then computed and considered as the mouse position within the cage. As shown in Figure 3, since the camera view is not orthogonal with respect to the cage floor (i.e., view from the top), the video frames are conveniently re-projected via an estimated homography so that distances are properly scaled [22]. Our video processing software has been developed by leveraging the OpenCV library in Python 3 [22]. To validate our video processing SW we compared its centroids estimation with the ones obtained with Ethovision XT 10 (see [23]). We randomly picked up 15000 video frames and compared the average absolute error between centroids coordinates, obtaining an average error of 3.8%.

### 2.3 Validation methods and data processing

To validate the proposed home cage monitoring system, we considered three cages, each with an individually housed mouse, and compared the activity metrics obtained with the CST-based system and video processing (see Section 2.4) while observing the cages simultaneously with both systems. Metrics comparisons are thus performed on the same animal subjects at the same time. Since the proposed system is capable of monitoring home cages 24/7, we decided to observe the cages for an entire week to capture activity cycles occurring over multiple days. To make data processing more tractable we organized data collection in intervals of 30 minutes of continuous recording for both systems (synchronized to each other, and referred to as *video block*). During each video block, the video camera produced approximately 18000 frames (10 frames/sec), while the CST board provided approximately 7200 measurements for each of the 12 electrodes (4 samples/sec).

Since the CST board used in the proposed home cage monitoring system is designed to track mice activity while on the cage floor, we considered only the mouse positions derived via the video tracking SW such that the centroid is on the cage floor, otherwise positions are not accounted for in the metrics computation (e.g., while mice hang on the food grid). In other words, the comparison of the activity metrics defined in Section 2.4 is to be considered related to the time spent by the mice on the cage floor.

#### 2.3.1 Metrics comparison

To compare the metrics derived through the two systems we considered the Pearson correlation coefficient [10]. The correlation coefficient *R* is a measure of the linear correlation between two variables and takes values between −1 and 1, where R=1 indicates maximum positive correlation. The correlation coefficient also allows comparison between metrics that represent different physical quantities, which can thus be used to, e.g., compare distance walked with electrode activations (see metric definition in Sec. 2.4).

### 2.4 Mice activity metrics

In this paper, we consider activity metrics that are conventionally used in assessing animal activity in individually-housed mice (see, e.g., [14]), such as *distance walked*, *average speed* and *occupation* (rear and front). We also introduce a new metric, referred to as *activation density*, which is inspired by the capacitance sensing technology discussed in this paper. Distance walked and average speed are common metrics used in automated home cage monitoring (see e.g., [23]), while cage area occupation can be used to monitor, e.g., spatial preferences of mice (see e.g., [24]). Activation density instead, is a method of measuring animal activity that is specifically inspired by the CST discussed in this paper, even though similar metrics have been used with different sensing technologies, see e.g., [8]. A deeper analysis of literature on metrics and methods describing animal activity can be found in [3].

#### 2.4.1 Distance walked and average speed

The distance walked accounts for the total distance covered by the mouse within a given time interval, while the average speed is the distance walked divided by the duration of the time interval considered. We assume that the mouse position on the cage floor is identified in terms of its centroid, while the distance walked is computed via the sum of the euclidean distances of the mouse centroid in successive frames within the time interval of interest. The distance walked is defined as follows. Let $p(t)=[p_{x}(t),p_{y}(t)]$ be a 2×1 vector of coordinates on the plane (cage floor) representing the position of the centroid of the mouse at time *t*. Then, the distance walked within the time interval $t_{1}$ and $t_{2}$ can be computed as

(1) $$S(t_{1},t_{2})=\sum_{t=t_{1}+1}^{t_{2}}d(t),$$

where

(2) $$d(t)=\sqrt{{\left(p_{x}(t)-p_{x}(t-1)\right)}^{2}+{\left(p_{y}(t)-p_{y}(t-1)\right)}^{2}},$$

is the euclidean distance between two positions in adjacent frames. Definition (1) is used for both the proposed capacitance sensing-based system and video-based distance measurements. The average speed is instead defined as the ratio between the cumulative walked distance (1) and the duration of the time interval:

(3) $$V(t_{1},t_{2})=\frac{1}{t_{2}-t_{1}}S(t_{1},t_{2}).$$

The mouse position via video processing is derived as described in Section 2.2.1, whereas for the proposed CST the mouse position (its centroid) is computed by conveniently weighing capacitance variations across the 12 electrodes of the CST board.

#### 2.4.2 Front/rear occupation metric

The *front (rear) occupation* metric measures the relative time spent by the mouse in the front (rear) part of the cage with respect to the total time in the cage. More specifically, let $T_{F}(n)$ be the time spent in the front part of the cage in the *n*th video block and let $T_{B}(n)$ be the duration of the *n*th video block (say measured via video tracking system, but the same applies for capacitance sensing). Then the relative time spent in the front part of the cage (*front occupation*) is given by $T_{F}(n)/T_{B}(n)$. Similarly we can compute the relative occupation in the rear part of the cage. The front and rear parts cover approximately one third of the cage floor area each. The total floor area is approximately 520 cm^2^, and thus one third corresponds to approximately 173 cm^2^.

#### 2.4.3 Activation density metrics

The *activation density* metric is inspired by the physical structure of the CST used by the proposed home cage monitoring system. An electrode is considered *activated* when its measurements are perturbed significantly over a limited time interval, which generally occurs when a mouse performs activity while sufficiently close to an electrode (see below). Density indicates that the total number of activations are divided by the duration of the time interval considered and the number of electrodes of interest (up to twelve). A sketch of the CST activation density metric is the following. Recall that the CST board provides measurements related to electrode capacitance every 250 ms and let $c_{k}(t)$ be the (filtered) capacitance measurements from the *k*th electrode at time *t*. Then, we compare the difference between two adjacent capacitance measurements as

(4) $$\Delta_{k}(t)=c_{k}(t)-c_{k}(t-1).$$

The rationale behind (4) is that when no animal movements occur the difference $\Delta_{k}(t)$ is approximately zero as there are no variations in electrode capacitance, while absolute values $\left|\Delta_{k}(t)\right|>0$ indicate capacitance variations that are generally caused by animal movements. According to these observations, we consider that an electrode is activated when we observe a changes in adjacent measurements larger than a fixed threshold *λ*. The threshold is conveniently chosen to separate noise induced capacitance variations from animal movements. Finally, the binary information indicating whether the electrode is activated at time *t* is given by

(5) $$a_{k}(t)=1[\left|\Delta_{k}(t)\right|\geq \lambda ],$$

where 1[x] is the indicator function for the event *x*, with 1[x]=1 if event *x* is true and 1[x]=0 otherwise.

Finally, one is generally interested in measuring the average amount of activations, occurring across a given set of electrodes (i.e., area of the cage) and within a given time interval. To do so, the CST *activation density* within time periods $t_{1}$ and $t_{2}$, across set of electrode S, can be computed as

(6) $$A_{CST}(t_{1},t_{2})=\frac{1}{\left|S\right|(t_{2}-t_{1})}\sum_{k\in S}\sum_{t=t_{1}}^{t_{2}-1}a_{k}(t),$$

where |S| indicates the cardinality (i.e., number of electrodes) of set S. Note that, the CST activation density does not indicate the type of movement performed, but it only accounts whether activity occurred close to an electrode.

Even though the activation density metric cannot be mapped directly to video analysis due to the different nature of the sensing mechanism (electrodes vs video frame pixels), we can define a video activation when a mouse (or better, its estimated centroid) moves between adjacent video frames for a distance larger than a given threshold ${\delta_{VID}}^{ACT}$ meters, and consequently, *video activation density* is obtained by dividing the total amount of video activation events by the number of video frames within the time interval considered. More specifically, the video activation density metric within time interval $t_{1}$ and $t_{2}$ is defined as:

(7) $$A_{VID}(t_{1},t_{2})=\frac{1}{\left(t_{2}-t_{1}\right)}\sum_{t=t_{1}}^{t_{2}-1}1\left[d(t)\geq \delta_{VID}^{ACT}\right],$$

where again 1[x] is the indicator function and d(t) is derived as in (4). In other words, we consider that an activation event occurred between two adjacent video frames when the centroid representing the mouse moves at least $\delta_{VID}^{ACT}$ meters.

### 2.5 Animals

Three individually housed C57BL/6J mice were observed non-intrusively while kept in their home cage (Tecniplast GM500) into an IVC rack, equipped with the CST-based home cage monitoring system introduced in this paper, at the University of Camerino (Macerata, Italy). Animal room was subject to a 12:12 h light-dark schedule (lights on at 09:00 am and lights off at 09:00 pm) with ad libitum food and water (auto watering system). All procedures were carried out in accordance with the recommendations of the European Community Council Directive and the National Institutes of Health for the Care and Use of Laboratory Animals and were approved by the University of Camerino Internal Ethical Committee for the Laboratory Animal Protection and Use.

## 3 Results

**Figure 4:**
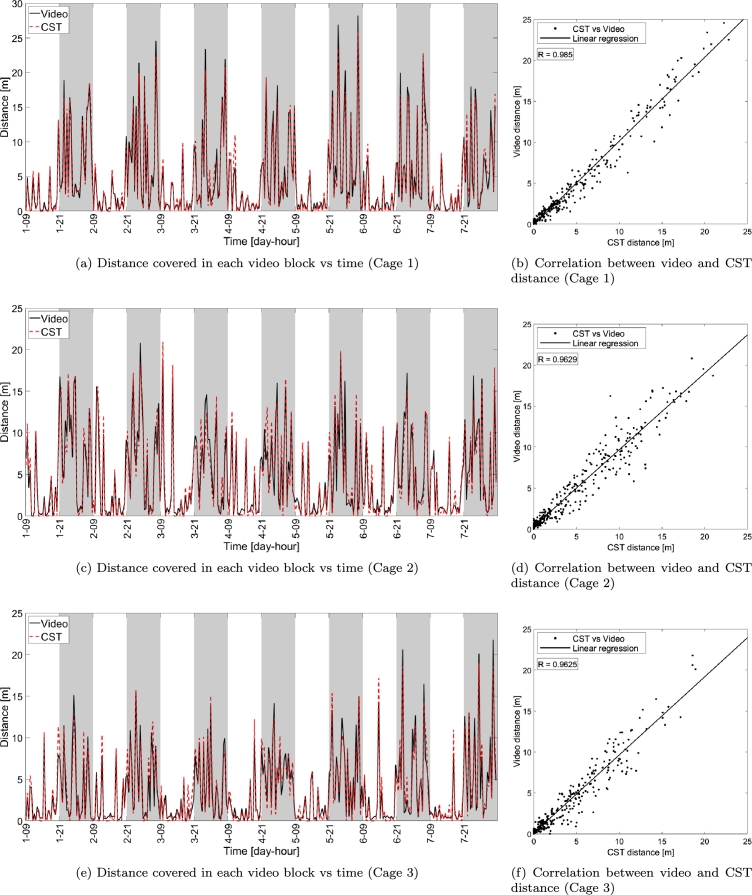
Distance computed via the proposed capacitance-based home cage monitoring system and video versus time (panels (a), (c) and (e)) and scatter plot comparing the two tracking systems (video on vertical axis and CST on horizontal axis) for the three cages under test (panels (b), (d) and (f)). The solid lines in the scatter plots indicate the linear fitting of the measurements while each dot corresponds to a single video block of 30 minutes. *R* indicates the correlation between the measurements obtained with CST and video. Gray shaded areas indicate dark periods (lights off).

**Figure 5:**
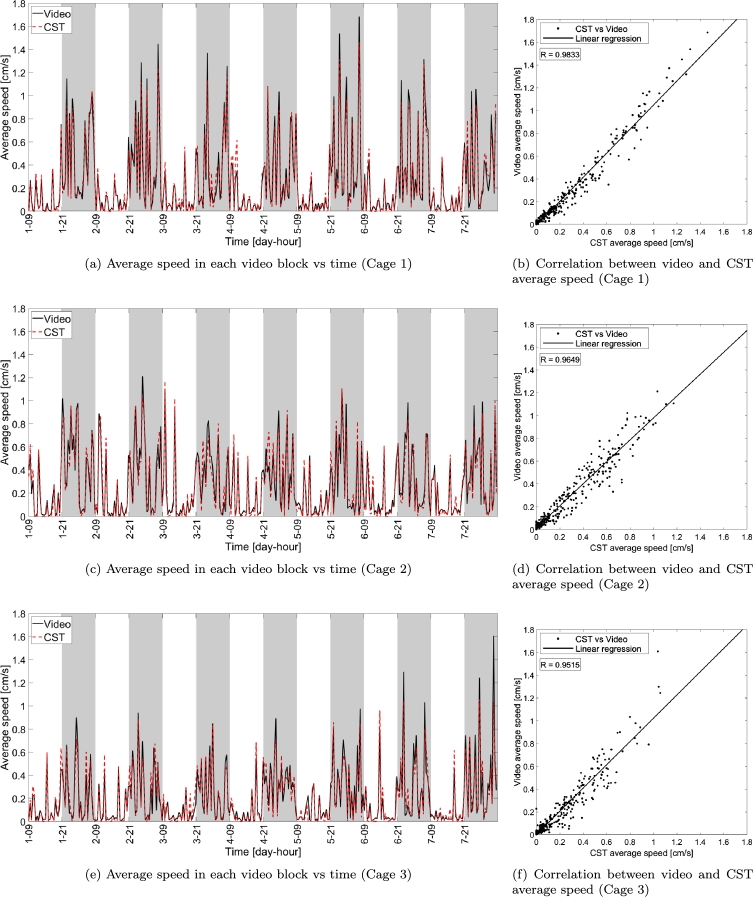
Average speed computed with CST-based system and video versus time (panels (a), (c) and (e)) and scatter plot comparing the two tracking systems (video on vertical axis and CST on horizontal axis) for the three cages under test (panels (b), (d) and (f)). The solid lines in the scatter plots indicate the linear fitting of the measurements while each dot corresponds to a single video block of 30 minutes. *R* indicates the correlation between the measurements obtained with CST and video. Gray shaded areas indicate dark periods (lights off).

The distances covered by the mouse in each video block measured with the proposed CST-based home cage monitoring system and video tracking (*CST distance* and *video distance* in short), respectively, are shown in the left panels (a), (c) and (e) of Figure 4 versus time for all the three cages, whereas correlation is analyzed via the scatter plots shown in panels (b), (d) and (f). The two metrics are highly correlated (the minimum correlation measured across the three cages is larger than R≥0.9625), indicating that the information provided by the two systems, in terms of distance walked by an individually-housed mouse, is very similar. The same considerations apply to the average speed (minimum correlation value R≥0.9515), whose plots are shown in Figure 5.

**Figure 6:**
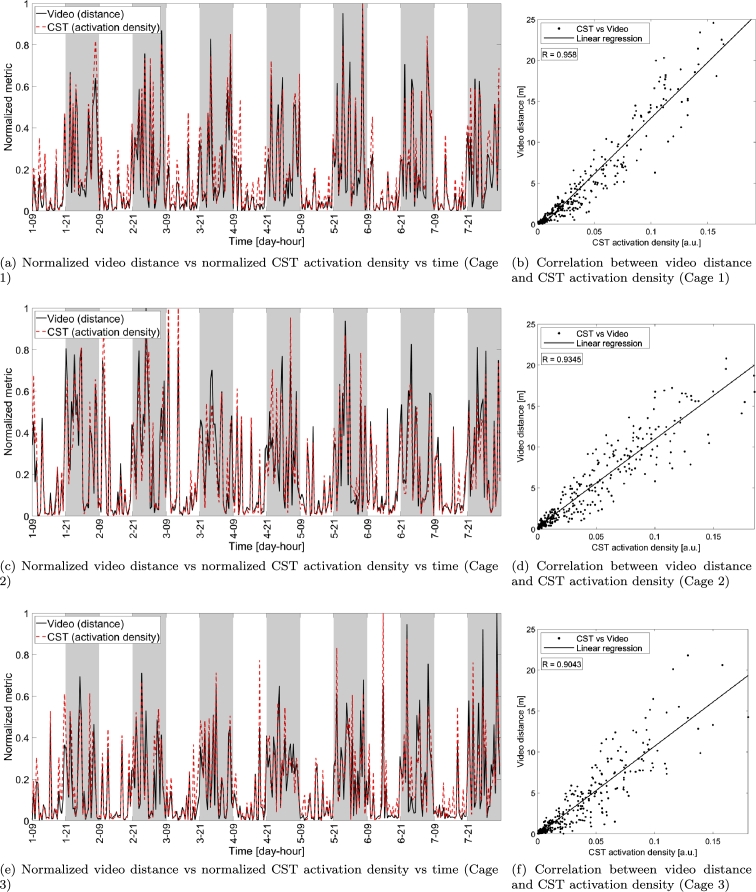
Comparison between video distance and activation density obtained with the proposed capacitance sensing technology (both normalized with respect to their maximum value in the week-long interval) in each video block versus time (panels (a), (c) and (e)) and scatter plot showing the correlation between the two metrics (video distance on vertical axis and CST activation density on horizontal axis) for the three cages under test (panels (b), (d) and (f)). The solid lines in the scatter plots indicate the linear fitting of the measurements while each dot corresponds to a single video block of 30 minutes. *R* indicates the correlation between the measurements obtained with CST and video.

We also assessed the CST activation density by comparing its measurements with video distance. Since activation density and distance are two distinct physical quantities we plotted them normalized for their maximum value within the week-long interval (see panels (a), (c) and (e) of Figure 6). The correlation is instead addressed in panels (b), (d) and (f) where metrics are not normalized (normalization is not necessary since Pearson correlation is scale invariant). The correlation values are smaller than the ones for video distance and CST distance (minimum correlation value was R≥0.9043) but indicate that CST activation density is highly related to mice locomotion measured via video tracking distance, even though they represent different physical quantities.

**Figure 7:**
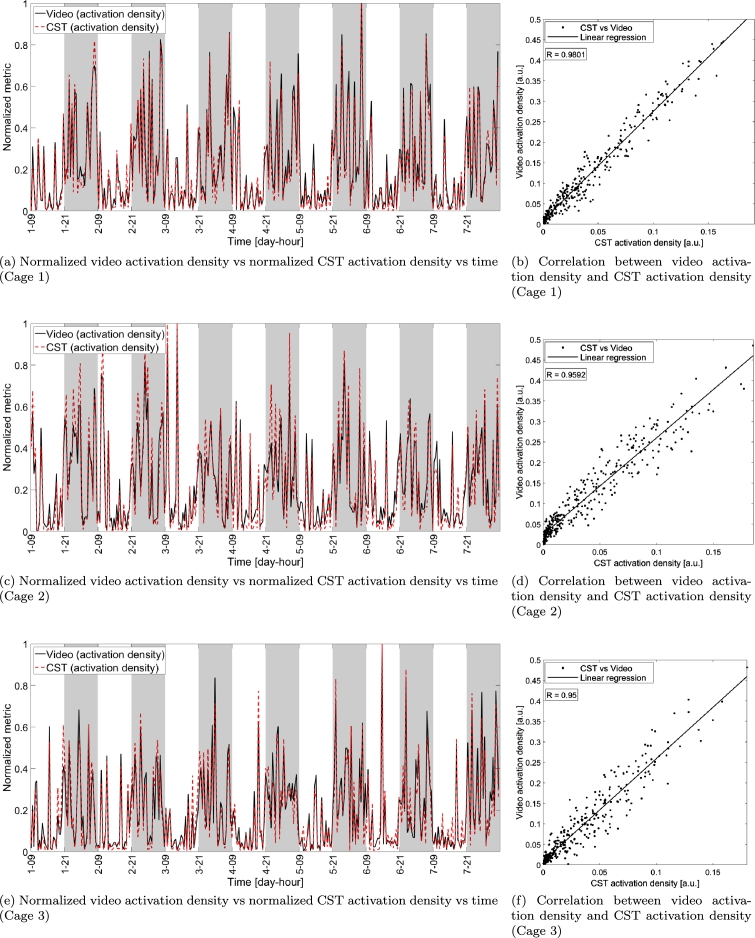
Comparison between video activation density and CST activation density (both normalized with respect to their maximum value in the week-long interval) in each video block versus time (panels (a), (c) and (e)) and scatter plot showing the correlation between the two metrics (video activation density on vertical axis and CST activation density on horizontal axis) for the three cages under test (panels (b), (d) and (f)). The solid lines in the scatter plots indicate the linear fitting of the measurements while each dot corresponds to a single video block of 30 minutes. *R* indicates the correlation between the measurements obtained with CST and video.

As discussed in Sec. 2.4.3, we introduced video activation density as a metric to indicate when a mouse moves a distance larger than a given threshold $\delta_{VID}^{ACT}≃1\text{ mm}$. The correlation between the video activation density and the CST activation density (see Figure 7) indicate that the two metrics are highly correlated (minimum value R≥0.95), meaning that, even though they are measured with different systems and metrics, the information provided are highly related to each other.

**Figure 8:**
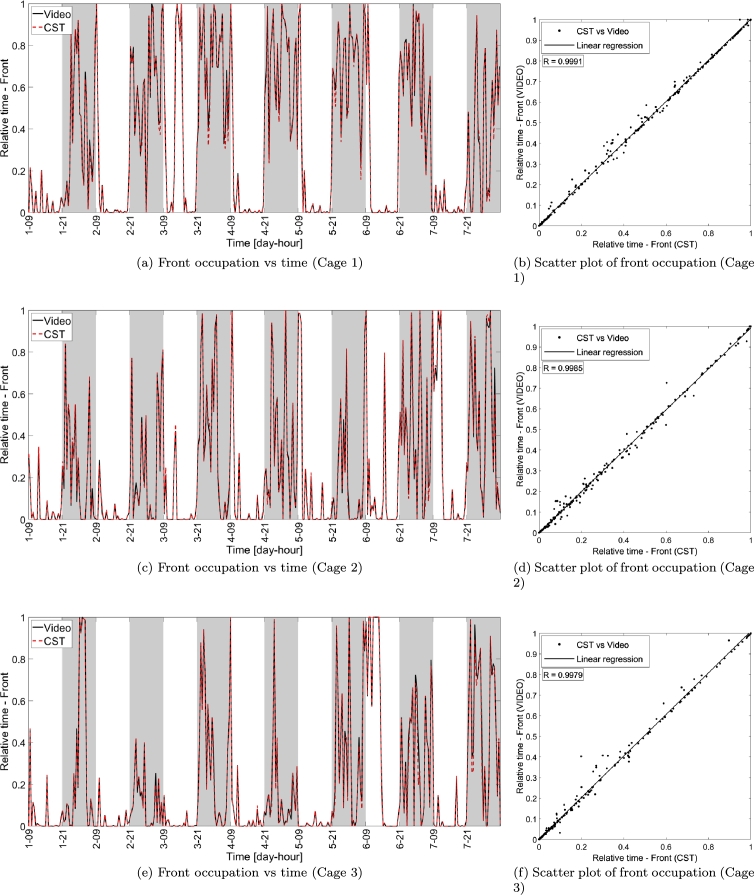
Relative time spent in the front part of the cage measured via video and CST, respectively. Panels (a), (c) and (e) show the front part occupation versus time in the week-long interval considered. Panels (b), (d) and (f) show the scatter plot of the CST and video measurements, where each data point corresponds to a single video block of 30 minutes, while the black solid line indicates the linear regression of the measurements, while *R* indicates the correlation between the measurements obtained with CST and video.

**Figure 9:**
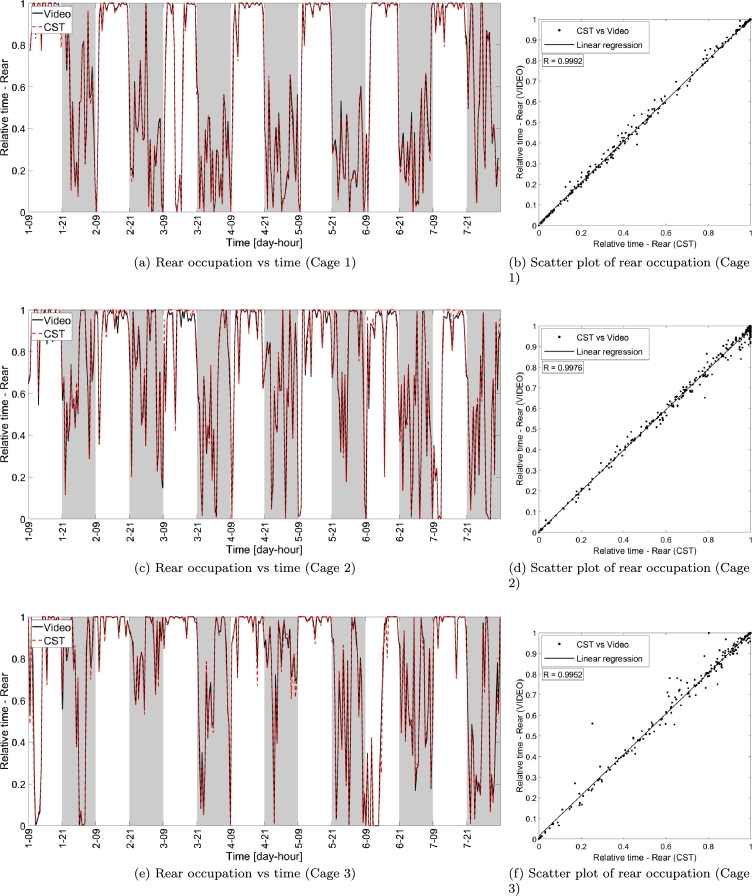
Relative time spent in the rear part of the cage measured via video and CST-based system, respectively. Panels (a), (c) and (e) show the rear part occupation versus time in the week-long interval considered. Panels (b), (d) and (f) show the scatter plot of the CST-based and video measurements, where each data point corresponds to a single video block of 30 minutes, while the black solid line indicates the linear regression of the measurements, while *R* indicates the correlation between the measurements obtained with CST-based system and video.

The comparisons between cage occupancy (see Sec. 2.4.2) measured with CST and video are instead addressed in Figure 8, where panels (a), (c) and (e) show the occupation in terms of the fraction of time spent in the front part of the cage (with respect to the total duration of each video block) for the three cages under test. The correlation between the metrics provided by the two systems (see panels (b), (d) and (f), of Figure 8), show values approaching unity, thus indicating that the cage occupancy derived with CST and video are very similar for all video blocks considered. Similar considerations apply for the rear part of the cage as shown in Figure 9.

**Figure 10:**
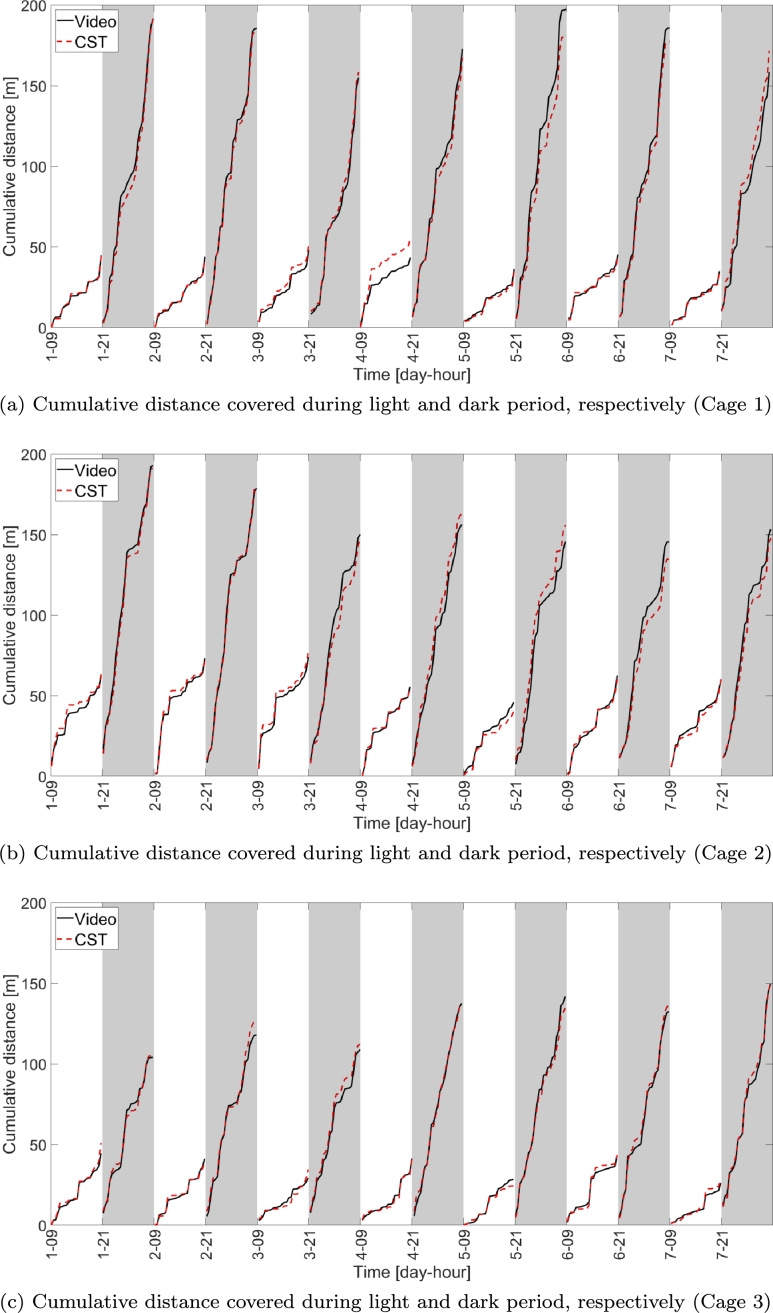
Cumulative distance computed via CST-based home cage monitoring system and video versus time for the three cages under test. Covered distance is cumulated over each light and dark period, so that the last point within each period indicates the total distance covered with the corresponding period.

The cumulative distance covered by each mouse during each light and dark period, respectively, is shown in Figure 10. The cumulative distance is obtained by incrementally summing up each 30 minutes video block within each light/dark period (up to 24 video blocks of 30 minutes within a 12 hours period), so that the last point within each period corresponds to the total distance walked in that period.

### 3.1 Results summary

Overall, all the activity metrics considered are highly correlated when comparing results obtained with the proposed CST-based home cage monitoring system and video processing (see Table 1 for a summary of the average correlations across the three cages under test). Clearly, correlations are higher when comparing the same physical quantities derived with CST and video (distance, average speed and occupation), while they are slightly lower when comparing distance with CST activation density, or video activation density with CST activation density. It is worth to mention that, a crucial role in estimating the trajectory of the mouse with both CST and video tracking is played by the smoothing approach of choice [25]. Trajectory smoothing include, e.g., filtering out small animal movements and reducing impact of noise. The effect of smoothing is strictly related to the technology in use, since for video we have a grid of 800x600 pixels, whereas for CST we have a grid of 4x3 electrodes, both used to cover the same cage floor (i.e., the resolution is not the same). In addition, the information extracted from video processing is also impacted by the camera positioning, which in our set up was placed just above the cage top at approximately 45° with respect to the center of the cage floor. As discussed in Sec. 2.2.1, this camera positioning required to apply an homography to each video frame to obtain proper distances walked on the cage floor. Even though this camera positioning is not optimal to derive the activity metrics considered in this paper (a top view would introduce less distortion to the images), we traded-off non optimal camera position with the possibility to monitor animals within their home cage in normal conditions and without requiring the use of special cage top nor the removal of the food holder. Nevertheless, we have shown that two completely different technologies, with independent processing algorithms, provide activity metrics that are highly correlated when observing the same subjects simultaneously.

**Table 1:** Summary of (Pearson) correlations between video and capacitance-sensing metrics.

VIDEO metric	CST metric	Average correlation
Distance	Distance	0.9701
Distance	Activation density	0.9323
Average speed	Average speed	0.9666
Occupation front	Occupation front	0.9985
Occupation rear	Occupation rear	0.9973
Activation density	Activation density	0.9631

## 4 Discussion & conclusions

In this paper we described a novel home cage monitoring system based on capacitance sensing technology (CST), which is able to non-intrusively monitor animal activity 24/7 while keeping home cages in the home rack and without impacting animal life. The main sensing capability of the proposed home cage monitoring system is provided by an electronic board placed underneath the home cage. Each board is equipped with 12 electrodes that exploit a proximity sensing technology to measure the capacitance of the environment surrounding each electrode. By monitoring capacitance variation over time, we have shown that it is possible to monitor animal activity and determine location of the mouse within the home cage. The activity metrics considered in this paper are distance walked, average speed, activation density and front/rear occupation. The metrics derived by processing capacitance measurements have been compared against the same (or similar) metrics derived via conventional video processing techniques. The comparisons have been conducted by simultaneously monitoring three individually-housed mice with both CST and camera-based systems and assessed in terms of Pearson correlation. High correlations have been found for both distance walked (average 0.9701) and average speed (average 0.9666) as well as for front occupation (average 0.9985) and rear occupation (average of 0.9973). We also introduced a new animal activity metric (activation density), specifically tailored to the capacitance sensing technology proposed in this paper, which shown high correlation values (average 0.9323) when compared against distance computed via video processing and even larger correlation when compared to video activation density (average 0.9631).

Even though this paper focuses on individually-housed mice as the initial step for the validation of the proposed CST-based home cage monitoring system, the activation density metric that we introduced in this paper can be generally extended to an arbitrary number of mice grouped in the same home cage. This has been actually already tested successfully in early applications of the proposed system [24], [26], [27] where activity of group-housed mice have been addressed. The derivation of new metrics as well as the validations for grouped mice are ongoing and will be subject of future investigations.

Finally, the proposed CST-based home cage-home rack monitoring system has been designed as a high throughput system that is capable of collecting 24/7 data from potentially thousands of home cages simultaneously (while keeping them on the home cage rack) with very limited network bandwidth and storage requirements, and without needing any human interaction once installed.

## Declarations

### Author contribution statement

Fabio Iannello: Conceived and designed the experiments; Analyzed and interpreted the data; Contributed analysis tools or data; Wrote the paper.

### Funding statement

This work was supported by Tecniplast SpA and the University of Camerino.

### Competing interest statement

The authors declare the following conflict of interests: Fabio Iannello is an employee of Tecniplast SpA. Tecniplast SpA provided support in the form of salary for the author Fabio Iannello, and provided all the devices and materials used to collect the datasets at the University of Camerino. Tecniplast SpA did not have any additional role in the study design, data collection and analysis, decision to publish, or preparation of the manuscript.

### Additional information

No additional information is available for this paper.

## Supplementary material

The following Supplementary material is associated with this article:

Activity metrics.zip Supplementary files containing activity and occupation metrics derived with DVC and Video processing. Activity metrics.zip
